# Mechanical Properties of Human Bronchial Epithelial Cells Expressing Wt- and Mutant CFTR

**DOI:** 10.3390/ijms21082916

**Published:** 2020-04-21

**Authors:** Ana P. Carapeto, Miguel V. Vitorino, João D. Santos, Sofia S. Ramalho, Tiago Robalo, Mário S. Rodrigues, Carlos M. Farinha

**Affiliations:** 1 BioISI – Instituto de Biosistemas e Ciências Integrativas, Faculdade de Ciências, Universidade de Lisboa, 1749-016 Lisboa, Portugal; apcarapeto@fc.ul.pt (A.P.C.); mvvitorino@fc.ul.pt (M.V.V.); jfsantos@fc.ul.pt (J.D.S.); ssramalho@fc.ul.pt (S.S.R.); ntrobalo@fc.ul.pt (T.R.); 2 Departamento de Física, Faculdade de Ciências, Universidade de Lisboa, 1749-016 Lisboa, Portugal; 3 Departamento de Química e Bioquímica, Faculdade de Ciências, Universidade de Lisboa, 1749-016 Lisboa, Portugal

**Keywords:** CFTR, AFM, Young modulus

## Abstract

Cystic fibrosis (CF) is caused by mutations in the gene encoding the cystic fibrosis transmembrane conductance regulator (CFTR). A single recessive mutation, the deletion of phenylalanine 508 (F508del), causes severe CF and resides on 70% of mutant chromosomes. Disorganization of the actin cytoskeleton has been previously reported in relation to the CF phenotype. In this work, we aimed to understand this alteration by means of Atomic Force Microscopy and Force Feedback Microscopy investigation of mechanical properties of cystic fibrosis bronchial epithelial (CFBE) cells stably transduced with either wild type (wt-) or F508del-CFTR. We show here that the expression of mutant CFTR causes a decrease in the cell’s apparent Young modulus as compared to the expression of the wt protein.

## 1. Introduction

The cystic fibrosis transmembrane conductance regulator (CFTR) is an integral membrane protein that belongs to the ATP-binding cassette (ABC) transporter superfamily and functions as a cyclic adenosine 3’,5’-monophosphate (cAMP)-regulated Cl-channel at the apical membrane of epithelial cells, including the respiratory epithelium [1]. CFTR plays an active role in controlling airway surface liquid homeostasis through the promotion of mucociliary clearance [2,3]. Mutations in the gene encoding CFTR cause the multiorgan disease Cystic Fibrosis (CF) [4], the most lethal recessive genetic inherited disorder in the Caucasian population [5]. The most common disease-causing mutation is the deletion of phenylalanine 508 (F508del), which leads to protein misfolding and intracellular retention.

CFTR membrane retention is regulated by several protein interactions, connecting it with the apical actin cytoskeleton, among which the Na^+^/H^+^ exchanger regulatory factor isoform-1 (NHERF-1) binds CFTR to the apical actin cytoskeleton and regulates CFTR membrane retention [6]. Therefore, the cytoskeleton integrity is critical for the immobilization of CFTR in the plasma membrane. Disease-causing mutations, such as F508del-CFTR, have been linked to the blocking of CFTR association with NHERF-1, resulting in the mis-targeting, altered recycling and impaired regulation of the channel [7]. The biochemical half-life of plasma membrane F508del-CFTR cells is about 4 h whereas the biochemical half-life of plasma membrane wt-CFTR exceeds 48 h [6,8]. In addition to the mediation of the association between CFTR and the cytoskeleton [9,10], overexpression of NHERF-1 in F508del-CFTR cells has been shown to stabilize the F508del-CFTR at the plasma membrane and to promote cytoskeleton organization [11]. Thus, CFTR membrane retention, CFTR association with NHERF-1 and cytoskeleton organization are key factors in CF disease mechanisms.

In the last decades, the use of Atomic Force Microscopy (AFM) to assess nanomechanical parameters—such as cell membrane elasticity—in correlation with cellular disease has increased [12,13]. AFM has been widely applied in single cell studies to analyse cellular elasticity, which is directly related to the structure of actin cytoskeleton filaments below the cellular membrane, which may be modified during the evolution of pathological conditions.

Generally speaking, two fundamental moduli can be used to characterize the mechanical behavior of an isotropic elastic material—the Young modulus and the Poisson ratio. The Young modulus (E) establishes a relationship between the force per unit area (stress) and the corresponding relative deformation (strain) in the elastic regime, that is, for small deformations. The stiffness of a material, which measures the resistance of an elastic body to deformation, besides depending on the sample geometry is directly proportional to its Young modulus. The Poisson ratio is defined as the ratio of transverse contraction strain to longitudinal extension strain in the direction of the stretching force. The Poisson ratio of cells is usually estimated to be 0.5, which means that cells are usually considered incompressible. It follows that the force necessary to indent a sample by a given amount is proportional to the quantity $E^{*}=E/\left(1-\nu^{2}\right)$, which we refer to as apparent Young modulus and is valid in the case where the indenter object has much larger Young modulus than the indented sample. In the case of soft matter (gels, polymers, biological material) where deformation under a given force is large, the assessment of mechanical properties is restricted to very small forces (nN or even pN range), which renders AFM one of the most suitable techniques for their study.

Based on force-indentation curves, AFM has been used to measure both elastic and viscous cellular responses, and a number of models have been proposed in an attempt to characterize the observed cellular behaviors. Although some models fit the experimental data quite well, most do not fully describe the observed behaviors and many appear contradictory in their predictions [14]. Each AFM approach-retract force curve gives information on the morphology and height of the sample, and by fitting data using one of the so-called contact models, it is possible to extract the sample Young modulus and adhesion force. Since the earliest AFM studies on soft biological samples, the prevalent methods used in the analysis of indentation data are based on the application of Hertz [15] and Sneddon [16] models of contact between two elastic bodies. The Hertz model describes the elastic deformation of spheres, whereas the Sneddon model accounts for other geometries such as conical or paraboloidal tips against a flat sample.

In this work, we performed a study of the mechanical properties of human bronchial epithelial cells stably transduced with either wt-CFTR or F508del-CFTR using a conventional AFM, and a custom-made Force Feedback Microscope (FFM) [17], which, unlike conventional AFM, measures indentation directly.

## 2. Results

**Figure 1:**
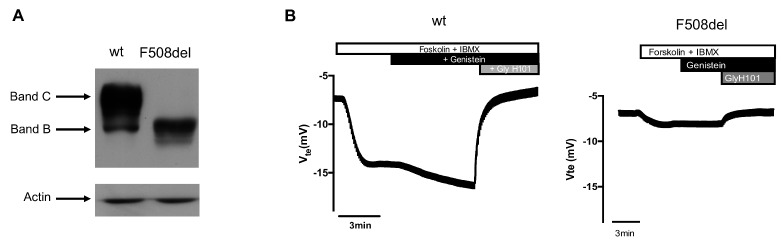
Biochemical and functional analysis of cystic fibrosis bronchial epithelial (CFBE) cells expressing wt- or F508del-cystic fibrosis transmembrane conductance regulator (CFTR). (**A**) Western Blot was used to assess the expression of CFTR in stably transduced CFBE cells and (**B**) transepithelial transport was assessed by Ussing chamber measurements of polarized CFBE cells expressing each variant.

We used cystic fibrosis bronchial epithelial cells transduced with wt- or F508del-CFTR [18]. Biochemical and functional characterization (Figure 1) confirm the lack of mature form and of forskolin-induced chloride transport in F508del-CFTR expressing cells as compared to those expressing wt-CFTR.

We have measured the apparent Young modulus using both AFM with a pyramidal tip and FFM with a spherical probe. For this purpose, cells were cultured on Petri dish supports with fibronectin-collagen coating solution, in liquid medium (phosphate buffer saline (PBS) buffer). Whenever referring to the apparent Young modulus we determine $E^{*}=E/1-\nu^{2}$, where *E* and ν are respectively the Young modulus and the Poisson ratio of the sample. We implicitly assume that the tip Young modulus is much larger than that of the sample.

### 2.1. Atomic Force Microscopy Indentation Tests

**Figure 2:**
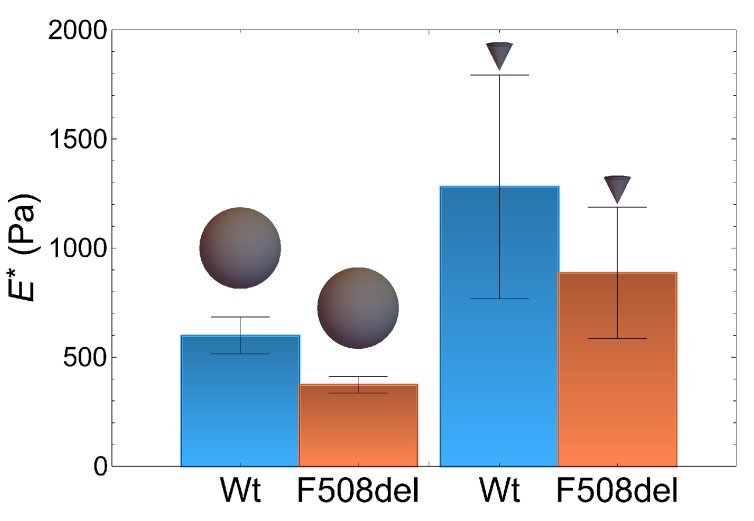
Atomic force microscopy analysis of wt-CFTR and F508del-CFTR cells obtained with Force Feedback Microscopy (FFM) using a spherical 1 μ m radius bead and with an Atomic Force Microscopy (AFM) and a pyramidal tip.

AFM apparent Young modulus results are presented in Figure 2 and were obtained for 1000 nm of indentation with a pyramidal tip. We have analysed 36 wt-CFTR cells and 32 F508del-CFTR cells. The apparent Young modulus of wt-CFTR cells is significantly larger than that F508del-CFTR cells, with 1280 (450) Pa and 886 (250) Pa median values and mean absolute deviation (MAD) in parenthesis respectively (interquartile ranges are 500 Pa and 300 Pa respectively). The datasets were compared using a Mann-Whitney test.

### 2.2. Force Feedback Microscopy Indentation Tests

The elasticity of wild type and F508del cells was additionally measured with Force Feedback Microscopy (FFM) [17,19,20] by performing indentation tests with a 1μ m radius bead. The elasticity trend obtained with the spherical probe agrees qualitatively well with that obtained by AFM using a much smaller probe radius. Wild type cells present higher values of elasticity as shown in Figure 2, 600 (130) Pa and 333 (75) Pa median values for wt-CFTR and F508del-CFTR respectively, with the MAD in parenthesis (interquartile ranges are 160 Pa and 75 Pa). However, the absolute values of elasticity obtained in the case of the pyramidal tip are larger than those obtained for the spherical tip by a factor slightly greater than 2. Here again the datasets were compared using a Mann-Whitney test (p<0.01 for both AFM and FFM data).

## 3. Discussion

CF is caused by the loss of the chloride channel function of CFTR. However, the CF underlying mechanism of this dysfunction is not fully understood. In this context, we performed a study using CFBE cells, initially from an individual with cystic fibrosis homozygous for the F508del CFTR. Since endogenous expression of CFTR was not detectable, this cell line was later transduced to express either wt- or F508del-CFTR—these two cell lines were analysed here by AFM and FFM. Cell stiffness has been described to be strongly affected by the actin filamentous structures under the cell membrane [21]. In recent years, force microscopy has unveiled the relevance of these nanomechanical properties in important cellular mechanisms, such as migration/locomotion [22], differentiation [23] or as a marker for disease progression [24,25]. It has become evident that these properties are fundamental to explain cell’s structure, evolution, and response to different stimuli, making AFM a potential tool for biomedical diagnosis and prognosis, with very promising results already obtained in the areas of cancer [26] or cardiovascular diseases [27].

We estimated Young modulus from AFM and FFM parameters in order to understand the basic physical parameters linked to the expression of these two variants. The mechanical properties measurements with AFM and FFM were obtained with the sample and the cantilever immersed in a liquid medium, in this case, Phosphate-Buffered Saline (PBS) buffer. The measurement in liquid medium confers many advantages such as the elimination of capillary forces, the reduction of Van der Waals’ forces between the tip and sample, and the possibility of studying cells in the osmolarity and ion concentrations of the solutions found in the human body. Fixing the cells to the coated dishes avoids artifacts, cell division and locomotion during the force-curves acquisition. The use of fixed cells instead of living ones, is adequate to this study as we aim to compare the differences between mechanical properties of wt-CFTR and F508del-CFTR CFBE cells in the same conditions. In addition, we performed FFM measurements to validate the Young modulus values.

The values that we obtained for the Young Modulus of the cells analysed are in the range of 0.2–2 kPa, which is in agreement with what was reported previously for micromechanical analysis of human cells’ actin or microtubule stiffness (0.1–1 kPa) [12]. The only previous study of mechanical properties of CFBE cells [28] reported values in the range of 40–130 MPa, which are very high for what has been commonly described for cultured cells, including human epithelial cells [29,30], and thus cannot be directly compared with the results obtained here. Significantly different median values were obtained for the apparent Young modulus of wt-CFTR and F508del-CFTR CFBE cells. We observed that wild type cells have higher Young modulus than the CFBE cells expressing mutant F508del-CFTR. This difference can be a consequence of a more disorganized actin cytoskeleton possibly due to a depolymerization of the actin network fibres below the cell membrane, related with the F508del mutation. The discrepancy between the results obtained with a spherical probe and those obtained with the pyramidal probe is very much in agreement with the results obtained in living Chinese hamster ovary cells [31]. It was hypothesized that this discrepancy was due to the fact that the much larger probe averages the elasticity over the cell, yielding an effective elasticity modulus while the sharper probe is more local, and hence displays a larger variability.

Herein, we have demonstrated a fundamental nanomechanical comparison between wt-CFTR and F508del-CFTR CFBE cells with two complementary force spectroscopy techniques. In the short term, it is difficult to envisage the direct application of these techniques in a clinical or diagnostic context, as they are a low throughput approaches that require considerable expertise. They would, however, be of great value in further elucidating the mechanisms related to CF pathology.

## 4. Materials and Methods

### 4.1. Cell Culture

CFBE41o- cells stably expressing wild-type(wt)-CFTR and F508del-CFTR were grown in Earle’s Minimum Essential Medium (EMEM) (Lonza, #BE12-611F, Basel, Switzerland) supplemented with 10% (*v/v*) fetal bovine serum (FBS) (Gibco, #10270-106, Waltham, MA, USA) and 5 μ g/mL puromycin (Sigma, #P8833, St. Louis, MO, USA). Tissue culture dishes (35 mm) were coated with fibronectin-collagen coating solution (100 μ g/mL Bovine Serum Albumin (BSA) (Sigma, #A9647, St. Louis, MO, USA), 10 μ g/mL collagen from rat tail tendon (Roche, #11179179001, Basel, Switzerland), and 10 μ g/mL human fibronectin (BD Biosciences, #354008, San Jose, CA, USA) prepared in Laboratory of Human Carcinogenesis (LHC) basal medium (Gibco, #12677, Waltham, MA, USA). Cells were seeded into coated dishes and growth until reaching more than 90% confluence (corresponding to a compact monolayer, but still with some minor gaps visible). Cells were washed 3 times on ice with cold PBS and were incubated for 10 min with 3% (*w*/*v*) paraformaldehyde (PFA) (Sigma, #252549, St. Louis, MO, USA) supplemented with 2% (*w*/*v*) sucrose (Aldrich, #S7903, St. Louis, MO, USA) at 4 ºC. Finally, cells were washed ten times at room temperature (RT) and then kept in PBS (Dulbecco’s Phosphate-Buffered Saline, Corning, 21-031-CV, Corning, NY, USA), also at RT during the AFM procedure. New coated dishes were used for each measurement day.

### 4.2. Atomic Force Microscopy

The mechanical properties of human bronchial cells were measured in liquid medium (PBS buffer) with AFM at room temperature. We used this approach to compare CFBE41o- stably transduced with either wt- or F508del-CFTR. The cells were analyzed with a PicoLE Molecular Imaging system from Agilent Technologies (Keysight Technologies, Inc., Santa Rosa, CA, USA). A MSNL-C Bruker cantilever, with nominal stiffness of 0.01 N/m and a nominal tip radius of 2 nm was used in all experiments. A new AFM tip was used for each sample/measurement day. To measure the mechanical properties of cells we perform grids of typically 32 × 32 approach/retract force-displacement curves in a range of 40 μ m. The AFM detector was calibrated by performing force-series curves on a glass substrate.

**Figure 3:**
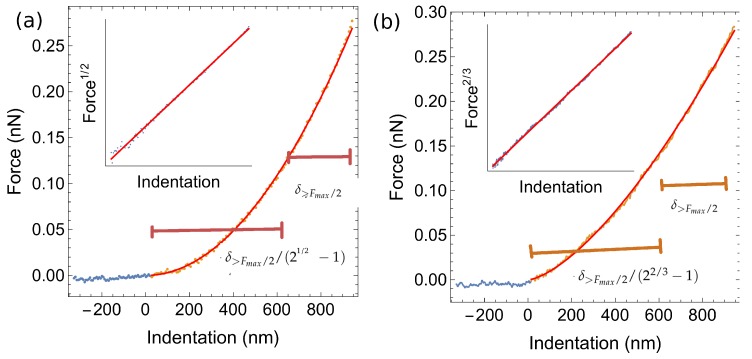
Force as a function of the indentation for (**a**) the pyramidal tip test, and (**b**) for the spherical bead test. The inset shows the force to the power of 1/2 (**a**) or 2/3 (**b**) as a function of indentation and the least squares fit to the data for the pyramidal tip (**a**) and conical tip (**b**) respectively.

For the determination of the Young modulus of the cell we used the Sneddon contact model [16] to fit the contact portion of the approach force curve. We assumed a conical indenter approximation (with nominal tip radius and an angle ≈21°). We have measured about 70 grids for two distinct conditions: A-CFBE cells expressing wt-CFTR and B- CFBE cells expressing F508del-CFTR. To fit the data to the Sneddon model, the following protocol was used: 1—the force base line corresponding to zero force was found; 2—the indentation was calculated by subtracting the cantilever deflection to the relative tip-sample separation; 3—the indentation span corresponding to forces above one half of the maximum applied load was determined; 4—the onset of contact was calculated by computing the total expected indentation span given by $\delta_{total}=\delta_{>F_{max}/2}+\delta_{>F_{max}/2}/\left(2^{1/2}-1\right)$; 5—selecting the region of the curve corresponding to $\delta_{total}$, the root square of the force was plotted as a function of the indentation; 6—a least squares method was used to fit the previous linear relationship. An example of this procedure is shown in Figure 3 where the resulting selection of points is shown in orange and in red a plot depicted with the values obtained from the linear regression described above. The inset shows the selected data points with the corresponding fit.

### 4.3. Force Feedback Microscopy

Concerning the measurements with the FFM, we used 1 μ m radius glass bead attached to cantilevers with nominal spring constant of 0.01 N/m. The indentation curves were limited to about 1 μ m.

Force Feedback Microscopy is an alternative to conventional AFM, which differs in the fact that an additional feedback loop is used to stabilize the cantilever position. Conventional AFM indentation tests involve the relative motion of the sample towards the cantilever tip, causing the cantilever to bend. To determine the indentation one must subtract the cantilever deflection (from which the force is calculated) from the relative tip-sample motion, which requires accurate calibration of the scanner motion and of the photodetector either via indentation experiments on hard samples or by measuring the cantilever spring constant using the Sader method [32] and then inferring the detection calibration from the cantilever thermal motion [33]. In FFM, the force is directly measured as a function of the indentation because there is no relative displacement of the tip. The measured force is the force necessary to maintain the tip at constant position and the force actuator is calibrated with an interferometric technique—an optical cavity is formed between the optical fiber extremity and the cantilever surface. Hence, the calibration of the force detector does not involve indentation on a different surface nor does it rely on the determination of the cantilever spring constant. To perform indentation tests, the sample is moved towards the cantilever, to which a sphere is attached. The force loop prevents motion of the bead by applying a counteracting force. The resulting measurement is directly the force versus indentation.

To analyse the curves, we performed a linearization by plotting the force to the power of 2/3 versus indentation. We proceeded as explained above for the AFM case with the following modifications—1—we do not need to calculate the indention because the data is measured directly as a function of indentation; 2—we calculate the total indentation span as $\delta_{total}=\delta_{>F_{max}/2}+\delta_{>F_{max}/2}/\left(2^{2/3}-1\right)$. An example of this linearization procedure is shown in Figure 3.

## Abbreviations

AFM	Atomic Force Microscopy
FFM	Force Feedback Microscopy
CF	Cystic fibrosis
CFBE	Cystic Fibrosis Bronchial Epithelial Cells
CFTR	Cystic fibrosis transmembrane conductance regulator
LD	linear dichroism

## References

[1] Stutts M.J., Canessa C.M., Olsen J.C., Hamrick M., Cohn J.A., Rossier B.C., Boucher R.C. (1995). CFTR as a cAMP-dependent regulator of sodium channels. Science.

[2] Boucher R. (2004). New concepts of the pathogenesis of cystic fibrosis lung disease. Eur. Respir. J..

[3] Li C., Naren A.P. (2005). Macromolecular complexes of cystic fibrosis transmembrane conductance regulator and its interacting partners. Pharmacol. Ther..

[4] Cheung J.C., Deber C.M. (2008). Misfolding of the cystic fibrosis transmembrane conductance regulator and disease. Biochemistry.

[5] Riordan J.R. (2008). CFTR function and prospects for therapy. Annu. Rev. Biochem..

[6] Farinha C.M. (2018). CFTR and Cystic Fibrosis. CFTR and Cystic Fibrosis.

[7] Voltz J.W., Weinman E.J., Shenolikar S. (2001). Expanding the role of NHERF, a PDZ-domain containing protein adapter, to growth regulation. Oncogene.

[8] Lobo M.J., Amaral M.D., Zaccolo M., Farinha C.M. (2016). EPAC1 activation by cAMP stabilizes CFTR at the membrane by promoting its interaction with NHERF1. J. Cell Sci..

[9] Ganeshan R., Nowotarski K., Di A., Nelson D.J., Kirk K.L. (2007). CFTR surface expression and chloride currents are decreased by inhibitors of N-WASP and actin polymerization. Biochim. Et Biophys. Acta (BBA) Mol. Cell Res..

[10] Okiyoneda T., Lukacs G.L. (2007). Cell surface dynamics of CFTR: The ins and outs. Biochim. Et Biophys. Acta (BBA) Mol. Cell Res..

[11] Favia M., Guerra L., Fanelli T., Cardone R.A., Monterisi S., Di Sole F., Castellani S., Chen M., Seidler U., Reshkin S.J. (2010). Na_+_/H_+_ exchanger regulatory factor 1 overexpression-dependent increase of cytoskeleton organization is fundamental in the rescue of F508del cystic fibrosis transmembrane conductance regulator in human airway CFBE41o-cells. Mol. Biol. Cell.

[12] Krieg M., Fläschner G., Alsteens D., Gaub B.M., Roos W.H., Wuite G.J., Gaub H.E., Gerber C., Dufrêne Y.F., Müller D.J. (2018). Atomic force microscopy-based mechanobiology. Nat. Rev. Phys..

[13] Kuznetsova T.G., Starodubtseva M.N., Yegorenkov N.I., Chizhik S.A., Zhdanov R.I. (2007). Atomic force microscopy probing of cell elasticity. Micron.

[14] Haase K., Pelling A.E. (2015). Investigating cell mechanics with atomic force microscopy. J. R. Soc. Interface.

[15] Hertz H., Jones D.E., Schott G.A. (1896). MiSCellane0us PaperS.

[16] Sneddon I.N. (1965). The relation between load and penetration in the axisymmetric Boussinesq problem for a punch of arbitrary profile. Int. J. Eng. Sci..

[17] Rodrigues M.S., Costa L., Chevrier J., Comin F. (2012). Why do atomic force microscopy force curves still exhibit jump to contact?. Appl. Phys. Lett..

[18] Bebok Z., Collawn J.F., Wakefield J., Parker W., Li Y., Varga K., Sorscher E.J., Clancy J. (2005). Failure of cAMP agonists to activate rescued ΔF508 CFTR in CFBE41o–airway epithelial monolayers. J. Physiol..

[19] Rodrigues M.S., Costa L., Chevrier J., Comin F. (2014). System analysis of force feedback microscopy. J. Appl. Phys..

[20] Vitorino M.V., Vieira A., Marques C.A., Rodrigues M.S. (2018). Direct measurement of the capillary condensation time of a water nanobridge. Sci. Rep..

[21] Kim S.O., Kim J., Okajima T., Cho N.J. (2017). Mechanical properties of paraformaldehyde-treated individual cells investigated by atomic force microscopy and scanning ion conductance microscopy. Nano Converg..

[22] van Helvert S., Friedl P. (2016). Strain Stiffening of Fibrillar Collagen during Individual and Collective Cell Migration Identified by AFM Nanoindentation. ACS Appl. Mater. Interfaces.

[23] Yang C., DelRio F.W., Ma H., Killaars A.R., Basta L.P., Kyburz K.A., Anseth K.S. (2016). Spatially patterned matrix elasticity directs stem cell fate. Proc. Natl. Acad. Sci. USA.

[24] Pyka-Fościak G., Zemła J., Lis G., Litwin J., Lekka M. (2020). Changes in spinal cord stiffness in the course of experimental autoimmune encephalomyelitis, a mouse model of multiple sclerosis. Arch. Biochem. Biophys..

[25] Hassan A.A., Vitorino M.V., Robalo T., Rodrigues M.S., Sá-Correia I. (2019). Variation of Burkholderia cenocepacia cell wall morphology and mechanical properties during cystic fibrosis lung infection, assessed by atomic force microscopy. Sci. Rep..

[26] Stylianou A., Lekka M., Stylianopoulos T. (2018). AFM assessing of nanomechanical fingerprints for cancer early diagnosis and classification: From single cell to tissue level. Nanoscale.

[27] Guedes A.F., Carvalho F.A., Malho I., Lousada N., Sargento L., Santos N.C. (2016). Atomic force microscopy as a tool to evaluate the risk of cardiovascular diseases in patients. Nat. Nanotechnol..

[28] Lasalvia M., Castellani S., D’Antonio P., Perna G., Carbone A., Colia A.L., Maffione A.B., Capozzi V., Conese M. (2016). Human airway epithelial cells investigated by atomic force microscopy: A hint to cystic fibrosis epithelial pathology. Exp. Cell Res..

[29] Takahashi A., Watanabe T., Mondal A., Suzuki K., Kurusu-Kanno M., Li Z., Yamazaki T., Fujiki H., Suganuma M. (2014). Mechanism-based inhibition of cancer metastasis with (-)-epigallocatechin gallate. Biochem. Biophys. Res. Commun..

[30] Luo Q., Kuang D., Zhang B., Song G. (2016). Cell stiffness determined by atomic force microscopy and its correlation with cell motility. Biochim. Et Biophys. Acta (BBA) Gen. Subj..

[31] Carl P., Schillers H. (2008). Elasticity measurement of living cells with an atomic force microscope: Data acquisition and processing. Pflügers Arch. Eur. J. Physiol..

[32] Sader J.E., Chon J.W., Mulvaney P. (1999). Calibration of rectangular atomic force microscope cantilevers. Rev. Sci. Instruments.

[33] Schillers H., Rianna C., Schäpe J., Luque T., Doschke H., Wälte M., Uriarte J.J., Campillo N., Michanetzis G.P. (2017). Standardized nanomechanical atomic force microscopy procedure (SNAP) for measuring soft and biological samples. Sci. Rep..

